# Rocuronium-induced anaphylaxis: a case report

**DOI:** 10.1186/s40981-019-0303-5

**Published:** 2019-12-06

**Authors:** Kanako Takahashi, Satoru Tanaka, Masanori Watanabe, Michiaki Yamakage

**Affiliations:** 1 Department of Anesthesiology, Oji General Hospital, 3-4-8 Wakakusacho, Tomakomai City, Hokkaido 053-8506 Japan; 2 Department of Anesthesiology, Sapporo Medical University School of Medicine, Minami 1-Jo Nishi 17-chome, Chuo-ku, Sapporo City, Hokkaido 060-8543 Japan

**Keywords:** Rocuronium, Vecuronium, Anaphylaxis, Skin prick test

## Abstract

**Background:**

Neuromuscular blocking agents are frequently a cause of anaphylaxis that occurs in the perioperative period, and a skin prick test is an examination for definite diagnosis.

**Case presentation:**

We report our experience of a patient with rocuronium-induced anaphylaxis who was scheduled to undergo open-heart surgery. After induction of anesthesia, anaphylaxis was suspected because the patient’s blood pressure decreased, airway pressure increased, and skin flushing and edema were observed on her neck and arms. With rapid treatment, good progress was seen without complications. About 5 weeks later, skin prick tests were performed for rocuronium and vecuronium. She was positive for rocuronium and negative for vecuronium. Seven weeks after anaphylaxis, vecuronium was used for the surgery and she had no symptoms that indicated anaphylaxis. The operation was completed uneventfully.

**Conclusion:**

We experienced a case of anaphylaxis caused by rocuronium. After a definite diagnosis had been made by a skin prick test, safe anesthesia management was possible using vecuronium during the reoperation.

## Background

The frequency of perioperative anaphylaxis is thought to be about one case in 10,000–20,000 worldwide [1]. According to the Medical Accident Investigation and Support Center of the Japan Medical Safety Research Organization, in recent statistics on population dynamics, the number of deaths due to anaphylaxis is about 50 to 80 per year and the most common cause is medicine (about 20 to 40 deaths per year) [2]. The drugs that most frequently cause anaphylaxis during general anesthesia are neuromuscular blocking agents (NMBAs) [1]. A case of multiple cross-reactivities to various NMBAs has been reported [3]. A skin test is used for a definite diagnosis of anaphylaxis [1].

We experienced a case of anaphylaxis caused by rocuronium. After a definite diagnosis had been made by a skin prick test, safe anesthesia management was possible using vecuronium during surgery that was performed 7 weeks later.

## Case presentation

Informed consent was obtained from the patient for publication of this case report and any accompanying images. A 74-year-old woman (body weight, 48 kg; height, 148 cm) without a history of drug allergy was scheduled to undergo open-heart surgery. She was taking oral medication for high blood pressure and atrial fibrillation. After hospitalization due to heart failure, severe mitral regurgitation and tricuspid regurgitation were found by echocardiography, and mitral valve replacement, tricuspid annuloplasty, and the maze procedure for atrial fibrillation were scheduled. Laboratory data were unremarkable except NT-proBNP 1920 pg/ml.

General anesthesia was induced with 4 mg of midazolam, 200 μg of fentanyl and 50 mg of rocuronium.

Tracheal intubation was performed uneventfully. Immediately after inserting a probe for recording a transesophageal echocardiogram, increase in airway pressure up to 40 cmH_2_O, reduction in blood pressure, and skin flushing and edema on her neck and arms were confirmed. Hate rate was 120 bpm or more and systolic arterial blood pressure fell to less than 60 mmHg and a low level persisted despite repeated administration of phenylephrine. An electrocardiogram showed no significant ST-T change in atrial fibrillation. With a possible diagnosis of anaphylaxis, we started chest compression and administered 1 mg adrenaline and 1000 mg methylprednisolone approximately 2 min after the onset of symptoms. In consideration of a possible latex allergy, the probe for a transesophageal echocardiogram was removed together with the probe cover, and the urinary catheter was also removed and replaced with a latex-free one. Following the insertion of a catheter into the right internal jugular vein, we started a continuous infusion of noradrenaline at 0.1 μg/kg/min. Although blood pressure and heart rate gradually stabilized approximately 30 min after starting treatment, the planned surgery was suspended. She remained orotracheally intubated and was transferred to the intensive care unit.

No further anaphylactic reaction or other complications occurred, and she was extubated the next day. Two days later, the results of drug-induced lymphocyte stimulation tests (DLSTs) for rocuronium and midazolam were negative.

**Fig. 1:**
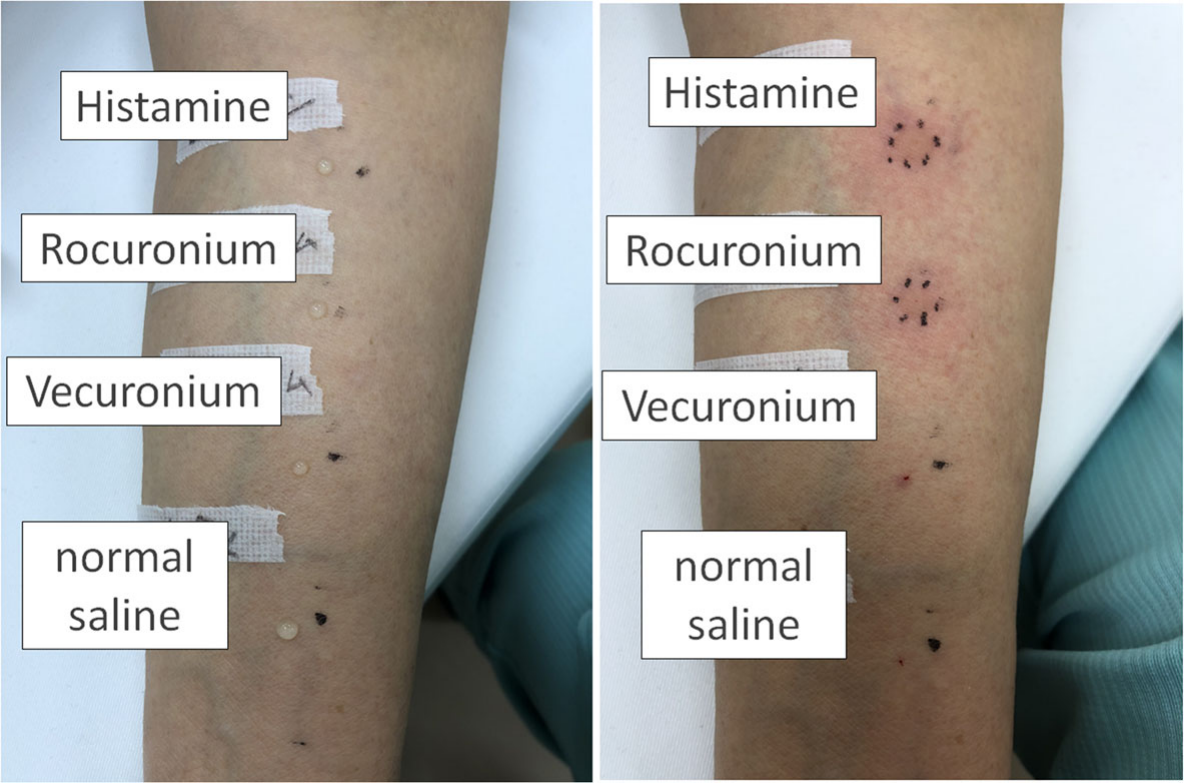
Skin prick test. From the top, in the order of histamine, rocuronium, vecuronium, and normal saline. The figure on the left is before puncturing, and the figure on the right is 15 min after puncture. Wheals are surrounded by black dots at histamine and vecuronium

Five weeks after anesthesia, skin prick test was conducted for rocuronium and vecuronium, following a method reported previously [1]. In brief, undiluted rocuronium and vecuronium (10 and 4 mg/ml, respectively), histamine (positive control) and normal saline (negative control) were prepared. One drop of the allergen was placed on the forearm flexion side, and the skin was punctured through the allergen with a 26 G needle. After 15 min, the diameter of the wheal (mean value of the longest diameter and the diameter perpendicular to the midpoint) was measured, and more than half of the positive control and 3 mm or more of the negative control were judged as positive [4]. The diameters of the wheals were 9 mm for histamine, 8 mm for rocuronium, and 0 mm for vecuronium and normal saline, and the results were therefore positive for rocuronium and negative for vecuronium (Fig. 1).

Surgery was performed seven weeks after anaphylaxis. General anesthesia was induced with midazolam, fentanyl, and vecuronium and was maintained with sevoflurane, vecuronium, and intermittent fentanyl. Surgery was completed uneventfully.

## Discussion

It has been reported that even without a history of general anesthesia, some people have IgE antibodies of NMBAs, suggesting the involvement of a quaternary ammonium structure common to muscle relaxants [1]. In our case, anaphylaxis occurred in the first general anesthesia.

Since the surgery was not emergent, it was suspended until definite identification of the antigen for anaphylaxis. Among various methods for diagnostic investigation, in vivo skin tests including prick and intradermal reaction tests remain the gold standard for detection of IgE-dependent allergies; these tests are best done after a delay of 4 to 6 weeks [1], when the antibody recovers to a sufficient level. We detected rocuronium, but not vecuronium, as an antigen by prick tests 5 weeks after the onset of anaphylaxis. On the other hand, a DLST 2 days after anaphylaxis was negative for rocuronium, probably due to its high false-negative ratio, particularly in the acute phase [5].

In our case, since a definite diagnosis was obtained by the skin prick test, no further examination was done, but a basophil activation test (BAT), which has been reported to have high levels of sensitivity and specificity as an in vitro examination, was also considered [6]. A limitation of this study is the lack of measurements of plasma histamine concentration and serum tryptase concentration immediately after the onset of anaphylaxis [1, 7].

According to the guidelines of the Japanese Society of Allergology, intramuscular injection of adrenaline is the first choice for the treatment of anaphylaxis [8]. Perioperative guidelines in several countries recommend intravenous injection, but there is no consensus regarding the intravenous dose [1]. In our case, since the anaphylactic shock was severe and the carotid artery became palpable, we administered 1 mg adrenaline intravenously.

## Conclusion

We experienced a case of anaphylaxis caused by rocuronium. After a definite diagnosis had been made by a skin prick test, safe anesthesia management was possible using vecuronium during the operation. BAT has been recognized as a promising tool for in vitro diagnosis of allergy or other hypersensitivity reactions.

## Data Availability

Not applicable due to patient privacy concerns.

## Abbreviations

BAT: Basophil activation test
DLST: Drug-induced lymphocyte stimulation test
NMBA: Neuromuscular blocking agent
